# Cervical Elastography as a Predictive Tool for Preterm Birth: A Systematic Review and Meta-analysis

**DOI:** 10.7759/cureus.92505

**Published:** 2025-09-17

**Authors:** Evgenia Angelopoulou, Kleanthi Gourounti, Angeliki Bolou, Maria Manesi, Athina Diamanti

**Affiliations:** 1 Department of Midwifery, Faculty of Health and Care Sciences, University of West Attica, Athens, GRC

**Keywords:** cervical elastography, diagnostic accuracy, preterm birth, shear wave elastography, strain elastography

## Abstract

Cervical elastography, including strain elastography (SE) and shear wave elastography (SWE), is an emerging ultrasound technique for assessing cervical stiffness, potentially enabling earlier prediction of spontaneous preterm birth than conventional sonographic measurements. However, its diagnostic performance and optimal application timing remain unclear. A systematic review and meta-analysis was conducted to evaluate the diagnostic accuracy of cervical elastography for predicting spontaneous preterm birth. Databases were searched for studies published between January 2014 and March 2025. Eligible studies assessed SE or SWE in pregnant women and reported sensitivity, specificity, and/or area under the receiver operating characteristic curve (AUC) for spontaneous preterm birth prediction. Data were extracted, and pooled estimates of sensitivity, specificity, diagnostic odds ratio (DOR), and AUC were calculated using a random-effects model. Subgroup analyses explored differences between SE and SWE and the influence of gestational age at assessment. Thirteen studies (n = 4,087 women) met the inclusion criteria. The pooled sensitivity was 77.1% (95% confidence interval (CI): 72.0-81.5), specificity 73.3% (95% CI: 66.4-79.3), and AUC 0.82 (95% CI: 0.78-0.85), with a pooled DOR of 11.05 (95% CI: 6.85-17.83). Subgroup analysis indicated that SWE tended to yield higher sensitivity in later gestation, whereas SE showed relatively better performance in early to mid-trimester assessments. Cervical elastography demonstrated moderate to good diagnostic accuracy for predicting sPTB. SE and SWE may have complementary roles depending on gestational age, supporting their potential integration into risk assessment strategies for preterm birth prevention.

## Introduction and background

Preterm birth, defined as delivery before 37 completed weeks of gestation, remains a leading cause of neonatal morbidity and mortality worldwide, accounting for over one-third of neonatal deaths and posing long-term neurodevelopmental and socioeconomic consequences [1]. It is classified into three subtypes based on gestational age: extremely preterm (<28 weeks), very preterm (28 to <32 weeks), and moderate to late preterm (32 to <37 weeks) [1]. Recent global estimates suggest that approximately 13.4 million babies were born preterm in 2020, with nearly 900,000 deaths in children under five years attributed to preterm birth-related complications in 2019 [1].

Despite advances in perinatal care, the global preterm birth rate remains high, estimated at ~10% of all live births, with significant variations between and within countries [2]. In the United States, a slight upward trend has been observed, with the rate rising from 10.1% in 2020 to 10.4% in 2022 [3].

The burden of preterm birth lies not only in its immediate perinatal consequences, such as respiratory distress syndrome, intraventricular hemorrhage, necrotizing enterocolitis, and neonatal sepsis, but also in its long-term sequelae, including neurodevelopmental disorders, feeding difficulties, and increased healthcare costs. The risk and severity of complications are inversely correlated with gestational age at delivery [3,4].

Current strategies to assess the risk of spontaneous preterm birth rely predominantly on transvaginal ultrasound measurement of cervical length (CL) and the detection of fetal fibronectin in cervicovaginal secretions [5,6]. However, these modalities show limited predictive performance, particularly in asymptomatic women without a prior history of preterm birth [7].

A growing body of evidence highlights that biomechanical and microstructural remodeling of the cervix, including collagen breakdown, changes in the extracellular matrix, and progressive softening of cervical tissue, precedes measurable shortening of the cervix [8,9]. These changes may remain undetected by conventional sonographic assessment, thereby necessitating the development of novel imaging modalities capable of evaluating tissue mechanical properties non-invasively and at an earlier stage [10].

Ultrasound elastography, an emerging imaging technique that quantifies tissue stiffness, has shown promise in this context. Two main approaches are currently in use: strain elastography (SE), which measures tissue deformation under applied pressure, and shear wave elastography (SWE), which evaluates stiffness based on the propagation speed of mechanically induced shear waves [11]. In obstetrics, cervical elastography has been proposed as a tool to identify women at high risk for spontaneous preterm birth, with the potential to detect early cervical softening even before shortening occurs [12]. Advanced systems using artificial intelligence have demonstrated improved diagnostic performance in asymptomatic populations [13].

Given the accumulation of new evidence, including studies employing novel elastographic platforms and incorporating machine learning algorithms, there is a pressing need to update the current synthesis of data. The present systematic review and meta-analysis aims to critically evaluate the diagnostic performance of cervical elastography in predicting spontaneous preterm birth.

## Review

Materials and methods

This systematic review and meta-analysis was conducted in accordance with the Preferred Reporting Items for Systematic Reviews and Meta-Analyses (PRISMA) guidelines [14] and followed the methodological framework for diagnostic accuracy reviews. The protocol was registered in the International Prospective Register of Systematic Reviews (PROSPERO; ID No. CRD420251119987).

Search Strategy

A comprehensive literature search was performed in PubMed, Embase, Web of Science, Scopus, and the Cochrane Library to identify relevant studies published from January 1, 2014, to June 30, 2025. The search combined MeSH terms and free-text keywords including the following: “cervical elastography”, “ultrasound elastography”, “shear wave elastography”, “strain elastography”, “preterm birth”, and “preterm delivery”. No language restrictions were applied during the initial search. Reference lists of included articles and relevant reviews were manually screened to identify additional eligible studies.

Inclusion and Exclusion Criteria

We included prospective cohort studies and case-control studies that (a) assessed cervical stiffness using ultrasound elastography (strain elastography (SE) or shear wave elastography (SWE)); (b) included pregnant women with or without symptoms of preterm labor; (c) reported preterm birth (<37 weeks of gestation) as an outcome; (d) provided sufficient data to construct 2 × 2 contingency tables (true positives (TP), false positives (FP), false negatives (FN), true negatives (NT)) for the prediction of spontaneous preterm birth; and (e) used a reference standard of clinical diagnosis of gestational age at delivery.

Exclusion criteria were reviews, editorials, conference abstracts without full data, non-human studies, and studies using elastography solely for labor induction prediction.

PRISMA process

Two reviewers independently screened titles and abstracts for eligibility. Full-text articles of potentially eligible studies were assessed against inclusion criteria. Disagreements were resolved by consensus or third-party adjudication.

The systematic search across five electronic databases yielded 1276 unique records (PubMed: 412; Embase: 389; Web of Science: 241; Scopus: 182; Cochrane Library: 52). A further 14 potentially relevant records were retrieved through manual screening of the reference lists of eligible articles and relevant reviews, bringing the total to 1290 records prior to duplicate removal.

Following the removal of 312 duplicate entries, 978 records proceeded to title and abstract screening. At this stage, 912 records were excluded because they did not meet the pre-specified inclusion criteria, most commonly due to irrelevance to the research question (e.g., absence of cervical elastography assessment, no preterm birth outcome reported, or unrelated clinical context).

The remaining 66 articles underwent full-text review to determine final eligibility. After detailed assessment, 53 studies were excluded for the following reasons: (a) insufficient diagnostic data to construct 2 × 2 contingency tables for sensitivity and specificity analysis (n = 21); (b) ineligible study design, including narrative reviews, editorials, or conference abstracts lacking complete data (n = 14); (c) non-human studies (n = 3); and (d) studies in which cervical elastography was used exclusively for predicting labor induction success, not spontaneous preterm birth (n = 15).

Ultimately, 13 studies fulfilled all eligibility criteria and were included in both the qualitative synthesis and the quantitative meta-analysis.

**Figure 1:**
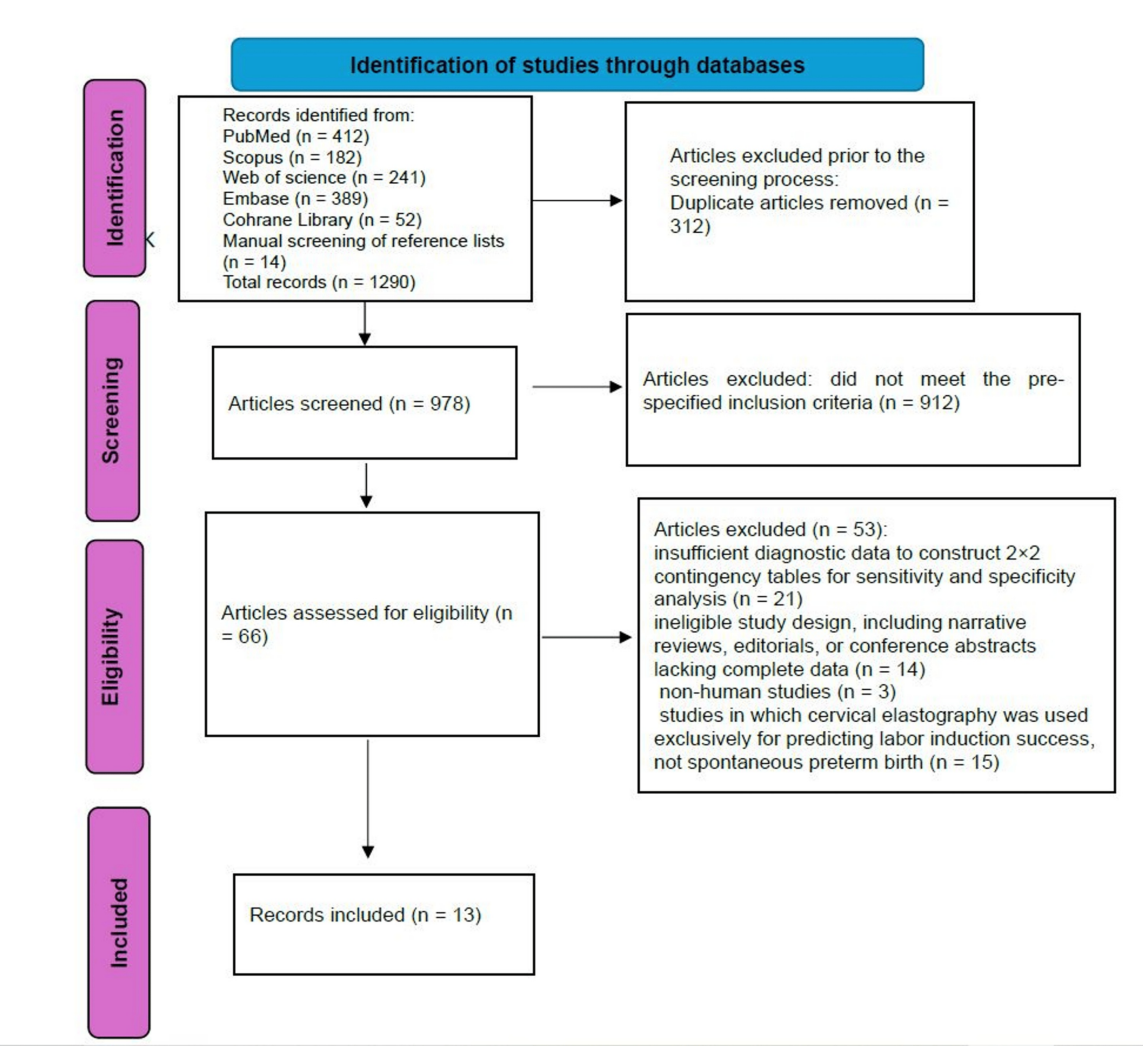
Flowchart of the study selection process.

The study selection process is illustrated in Figure 1.

Quality Assessment

The Quality Assessment of Diagnostic Accuracy Studies-2 (QUADAS-2) tool was used to evaluate the risk of bias and applicability across four domains: patient selection, index test, reference standard, and flow/timing [15]. Quality assessment results are available in Appendix 1.

Data Extraction

From each study, the following data were extracted: first author, year of publication, country, study design, sample size, prevalence of the primary outcome, diagnostic modality (SE vs. SWE), ultrasound approach (transvaginal vs. transabdominal), population type (symptomatic vs. asymptomatic), gestational age at assessment (second vs. third trimester), and performance metrics (area under the curve (AUC) with 95% confidence intervals (CIs), sensitivity, specificity). Where necessary, 95% CIs were used to back-calculate standard errors.

Outcomes

The primary outcomes were pooled sensitivity, pooled specificity, and pooled AUC for the index test in predicting preterm birth. Secondary outcomes included the positive likelihood ratio (LR+), negative likelihood ratio (LR−), and diagnostic odds ratio (DOR).

Statistical Analysis

Study-specific sensitivity and specificity were calculated from 2 × 2 contingency tables. Meta-analyses of proportions were conducted using the logit transformation to stabilize variances. For sensitivity and specificity, the standard error (StE) was calculated from the binomial distribution as:

StE(p) = √[ p × (1 − p) / n ]

where p is the proportion and n is the relevant denominator (TP + FN for sensitivity, TN + FP for specificity).

For the area under the curve (AUC), the standard error was derived from the reported 95% confidence interval (CI) using the formula:

StE = (Upper CI − Lower CI) / (2 × 1.96)

The variance of the logit-transformed AUC was estimated using the delta method:

Var(logit(p)) ≈ Var(p) / [ p² × (1 − p)² ]

Pooled estimates for each metric were obtained using a random-effects model (DerSimonian-Laird method) with inverse-variance weighting. Statistical heterogeneity was assessed using Cochran’s Q and quantified with I², with values >50% indicating substantial heterogeneity. Between-study variance was reported as τ².

Subgroup Analyses

To explore potential sources of heterogeneity, subgroup analyses were conducted by: (a) SE vs. SWE; (b) population type, symptomatic vs asymptomatic women; and (c) gestational age, second vs. third trimester. Each subgroup analysis used the same random-effects model, and both subgroup levels were displayed in the same forest plot for direct comparison.

Leave-One-Out Sensitivity Analysis

A leave-one-out (LOO) analysis was performed to assess the robustness of pooled estimates by sequentially omitting each study and recalculating the pooled sensitivity, specificity, and AUC. The studies whose removal resulted in the highest pooled estimate or the lowest heterogeneity (I²) for each metric were identified.

Software

All analyses were conducted in Python (v3.11) (Python Software Foundation, Wilmington, NC, USA) using pandas, numpy, and matplotlib for data handling and visualization. The meta-analytic calculations, transformations, and variance estimations were implemented with custom code following established statistical methods for diagnostic accuracy studies.

Results

Study Characteristics

A total of 13 studies published between 2014 and 2025 were included [16-28]. Sample sizes ranged from 34 to 1264. The prevalence of the primary outcome varied widely, from 4.7% to 55.3%, reflecting differences in study populations, settings, and outcome definitions. Most studies adopted a cross-sectional design. The majority used SE via a transvaginal approach, although several used SWE. Both symptomatic and asymptomatic populations were represented, and the gestational age at assessment ranged from the second to the third trimester.

Summary of the baselines characteristics of the included studies is given in Table 1. 

**Table 1:** Baseline characteristics of the included studies. AUC: area under the curve, aOR: adjusted odds ratio, ARFI: acoustic radiation force impulse, AUROC: area under the receiver operating characteristic curve, CI: confidence interval, CL: cervical length, DOR: diagnostic odds ratio, EI: elastography index, EOS: external Os, GA: gestational age, IN: internal os, IQR: interquartile range, IOS: internal Os, kPa: kilopascal, LR+ positive likelihood ratio, LR–: negative likelihood ratio, NPV: negative predictive value, OA: overall accuracy, OR: odds ratio, PPROM: preterm premature rupture of membranes, PPV: positive predictive value, PTB: preterm birth, PTD: preterm delivery, ROI: region of interest, sens: sensitivity, spec: specificity, sPTD: spontaneous preterm delivery, SR: strain ratio, SWE: shear wave elastography, TVS: transvaginal sonography, VTQ: virtual touch quantification, wks: weeks.

Citation/country	Study design	Population	Sample size (analyzed)	Ultrasound system	Elastography type	ROI/reference tissue	Outcome definition	Primary outcome prevalence	Diagnostic accuracy (all sPTD)		Conclusion
Köbbing et al. (2014) [16]/Germany	Prospective observational	Pregnant women at ~26 weeks' gestation, evaluated by transvaginal ultrasound elastography	182	Not specified	Strain elastography	Four regions of interest on the anterior cervical lip	Spontaneous preterm delivery before 37 weeks	11.9%	Sens 59%, spec 86%, OR 1.474 per 0.1 increase in Rselective (p = 0.002)		Cervical strain measurement with ultrasound elastography shows correlation with sPTD risk, with high specificity
Hernandez-Andrade et al. (2014) [17]/USA	Cross-sectional observational	Singleton pregnancies, asymptomatic women at 16–24 weeks’ gestation	189	Hitachi HI Vision 900, 8–4 MHz transvaginal	Strain elastography (Hitachi HI Vision 900, 8–4 MHz transvaginal probe)	Six ROIs: endocervix and entire cervix in sagittal, internal os, and external os views	Spontaneous preterm delivery < 37 weeks	11%	OR 0.17 (95% CI 0.03–0.9) and OR 0.20 (95% CI 0.04–0.9), respectively, after adjustment		Low strain (stiffer tissue) in internal os independently associated with markedly reduced sPTD risk
Wozniak et al. (2014) [18]/Poland	Prospective observational	Singleton pregnancies, asymptomatic women at 18–24 weeks’ gestation	337	Hitachi EUB-7500	Strain elastography	Internal os region/reference: anterior cervical lip near bladder wall	Spontaneous preterm delivery before 37 weeks	35/337 (9.9%)	Sens 71.4%, spec69.0%, PPV 32.3%, NPV 91.9%, LR+ 2.30, LR– 0.41, DOR 5.61		Strain elastography can be a useful adjunct to cervical length measurement in predicting sPTD, especially early cases
Woźniak et al. (2015) [19]/ Poland	Prospective observational	Pregnant women at 18–22 weeks with cervical length ≤ 25 mm	101	Not specified	Strain elastography (color map assessment)	Internal os region/color-coded stiffness (red, yellow, blue, purple)	Spontaneous preterm delivery before 37 weeks	45/109 (44.6%)	Sens 82.2%, spec 75.0%, PPV 72.5%, NPV 84.0%		Elastographic evaluation of the internal cervical os in women with short cervical length can improve prediction of PTD; warm colors (red/yellow) indicate higher risk
Agarwal et al. (2018) [20]/India	Prospective observational case–control	Pregnant women 28–37 weeks GA; preterm group: clinical PTL; term group: ≥37 weeks not in labor; exclusion: multiple gestations, severe maternal/fetal comorbidities	60	Acuson S2000 (Siemens Healthcare)	Shear wave elastography (ARFI / VTQ)	Anterior cervical wall close to internal os (depth < 80 mm)	Preterm birth (<37 weeks)	30/60 (50%)	Sens 96.7%, spec 87%		Shear wave elastography of antenatal cervix is a strong predictor of preterm birth, outperforming cervical length
Agarwal et al. (2018) [21]/India	Prospective observational	Primigravida women, 28–37 weeks GA, with symptoms suggestive of preterm labor; exclusions: ruptured membranes, advanced labor, prior preterm birth, cervical surgery, multiple gestation, polyhydramnios	34	Acuson S2000 (Siemens Healthcare)	ARFI-based shear wave elastography (VTQ) + elastography index (EI)	Anterior wall of internal os, depth < 80 mm	Preterm birth (<37 weeks)	14/34 (41.2%)	Sens 93%, spec 90%		Strain elastography provides good predictive accuracy for sPTD in asymptomatic mid-trimester women and may complement cervical length measurement
Hernandez-Andrade et al. (2018) [22]/USA	Prospective observational	Singleton pregnancies, asymptomatic women at 20–24 weeks GA; exclusions: fetal anomalies, maternal comorbidities, multiple gestations	628	Samsung Medison WS80A	Shear wave elastography	Internal os region, anterior cervical lip near bladder wall	Spontaneous preterm delivery before 37 weeks	43/628 (6.8%)	Sens 65%, spec 74%		Shear wave elastography at mid-trimester is a useful tool to predict sPTD, particularly when combined with cervical length measurement
Du et al. (2019) [23]/China	Prospective nested case–control	Low-risk, asymptomatic singleton pregnancies assessed at 11–14, 20–24, 28–32 weeks	553	Samsung WS80A with ElastoScan; E-Cervix tool	Strain elastography	Internal os, external os, and whole-cervix ROI (semi-automatic)	Spontaneous preterm delivery < 37 weeks	26/553 eligible (4.7%)	IOS-2: sens 72.73%, spec 64.23%, PPV 14.68%, NPV 96.53%, LR+ 2.03, LR– 0.42.
Luca et al. (2023) [24]/Romania	Prospective observational	Singleton pregnancies, high-risk for PTB (short cervix <2.5 cm or ≥2 risk factors), 18–24 weeks GA	114	GE Voluson E10	Strain elastography	Anterior & posterior lips of internal os/reference: anterior and posterior lips of external os	Spontaneous PTB <37 weeks	63/114 (55.3%)	Sens 85.71%, spec 84.31%, PPV 87.10%, NPV 82.69%		SR < 0.93 independently associated with increased PTB risk; combined model with cervical length + maternal risk factors improved AUROC to 0.938
Nguyen‐Hoang et al. (2024) [25]/Vietnam	Prospective observational	Unselected singleton and twin pregnancies	1264 (1143 singleton, 121 twin)	Philips EPIQ 7	Shear wave elastography	Internal os region/reference: external os posterior lip	Spontaneous PTB < 37 weeks	Singleton: 57/1143 (5.0%); Twin: 33/121 (27.3%)	Across gestation, CL shorter in sPTB (p < 0.001); significant at 21+0–24+6 w (p = 0.039) and 28+0–32+6 w (p < 0.001). CSWE lower in sPTB (p = 0.013); significant only at 11+0–15+6 w (p = 0.036).		Cervical softening (first trimester, 11+0–15+6 w) precedes cervical shortening (from late second trimester, ≥21+0 w) in sPTB. First-trimester CSWE may allow early identification of high-risk women
Vasudeva et al. (2024) [26]/India	Prospective observational	Asymptomatic pregnant women, 16–24 wks GA, with ≥1 high-risk factor for sPTD or CL < 2.5 cm	204	GE Voluson E-8	Strain elastography	Internal os (sagittal view), whole cervix (sagittal), internal os (axial)/stiffest area outside cervix (posterior/lateral, ligament insertion)	Delivery before 37 wks (after 26 wks), spontaneous labour onset or after PPROM; excludes indicated PTD	71/204 (34.8%)	Sens 52.11%, spec 60.9%, PPV 41.57%, NPV 70.44%, LR+ 1.33, LR– 0.79, DOR 1.69, OA 57.84%		The study concluded that TVS-guided cervical strain elastography is a better independent predictor of spontaneous preterm delivery than cervical length measurement in asymptomatic, high-risk women during the mid-trimester, with modest overall predictive accuracy but strong negative predictive value for early preterm birth, supporting its use as an adjunctive tool in high-risk settings, particularly in low- and middle-income countries to target monitoring and interventions effectively
Lu et al. (2024)[27]/China	Prospective observational	Singleton pregnancies, asymptomatic women at 20–24 weeks GA	176	Mindray Resona 8	Shear wave elastography	Internal os region/reference: posterior cervical lip	Spontaneous preterm delivery before 37 weeks	16/176 (18.2%)	Sens 71.4%, spec 69.3%, PPV 34.2%, NPV 91.3%, LR+ 2.33, LR– 0.41, DOR 5.68		Lower cervical stiffness significantly associated with higher risk of sPTD (aOR 0.26, 95% CI 0.11–0.59)
Kiefer et al. (2025) [28]/Germany	Prospective cohort	Singleton pregnancies, asymptomatic women at 18–24 weeks GA	245	GE Voluson E10	Shear wave elastography	Internal os region/reference: posterior cervical lip	Spontaneous preterm delivery before 37 weeks	44/245 (18%)	Sens 73.3%, spec 69.0%, PPV 35.9%, NPV 92.3%, LR+ 2.36, LR– 0.39, DOR 6.05		Lower cervical stiffness values independently predicted sPTD (aOR 0.28, 95% CI 0.13–0.59)

Overall Diagnostic Accuracy

Table 2 summarizes the diagnostic performance metrics from included studies, including AUC (95% CI) and 2 × 2 table parameters.

**Table 2:** Summary of diagnostic performance metrics from included studies, including AUC (95% CI) and 2 × 2 table parameters. AUC: area under the curve, CI: confidence interval, FN: false negatives, FP: false positives, TN: true negatives, TP: true positives.

Study	Year	AUC	AUC 95% CI	TP	FP	FN	TN
Köbbing et al. [16]	2014	0.79	0.65-0.93	10	18	7	108
Hernandez-Andrade et al. [17]	2014	0.84	0.68-0.93	20	118	1	50
Wozniak et al. [18]	2014	0.9	0.87-0.93	30	8	5	290
Wozniak et al. [19]	2015	0.85	0.72-0.98	37	14	8	42
Agarwal et al. [20]	2018a	0.91	0.83-0.99	29	4	1	26
Agarwal et al. [21]	2018b	0.92	0.85-0.99	13	2	1	18
Hernandez-Andrade et al. [22]	2018	0.88	0.81-0.95	15	150	16	447
Du et al. [23]	2019	0.73	0.67-0.78	19	190	7	337
Luca et al. [24]	2023	0.85	0.79-0.86	54	8	9	43
Nguyen-Hoang et al. [25]	2024	0.9	0.87-0.93	66	369	24	805
Vasudeva et al. [26]	2024	0.62	0.58-0.64	37	52	34	81
Lu et al. [27]	2024	0.89	0.80-0.98	11	49	5	111
Kiefer et al. [28]	2025	0.6	0.59-0.61	32	62	12	139

The pooled diagnostic performance across all studies using a random-effects model showed a sensitivity of 0.771 (95% CI: 0.686-0.856), specificity of 0.733 (95% CI: 0.631-0.836), and an AUC of 0.820 (95% CI: 0.728-0.913). The positive likelihood ratio (LR+) was 3.056 (95% CI: 2.249-4.152), the negative likelihood ratio (LR-) was 0.342 (95% CI: 0.241-0.483), and the DOR was 11.052 (95% CI: 5.484-22.272). Statistical heterogeneity was high for sensitivity (I² = 85.5%), specificity (I² = 98.4%), and AUC (I² = 98.7%), indicating substantial between-study variability.

Table 3 summarizes the pooled sensitivity, specificity, likelihood ratios, DOR, and AUC, using a random-effects model.

**Table 3:** Pooled diagnostic accuracy meta-analysis results. AUC: area under the Curve, CI: confidence interval, DOR: diagnostic odds ratio, I²; inconsistency index (percentage of variability due to heterogeneity), LR+: positive likelihood ratio, LR–: negative likelihood ratio, Tau²: between-study variance.

Metric	Pooled	Lower 95% CI	Upper 95% CI	Tau²	I² (%)
Sensitivity	0.771	0.686	0.856	0.02	85.5
Specificity	0.733	0.631	0.836	0.034	98.4
LR+	3.056	2.249	4.152	0.251	91.2
LR−	0.342	0.241	0.483	0.262	77.5
DOR	11.052	5.484	22.272	1.29	86.1
AUC	0.82	0.728	0.913	0.027	98.7

**Figure 2:**
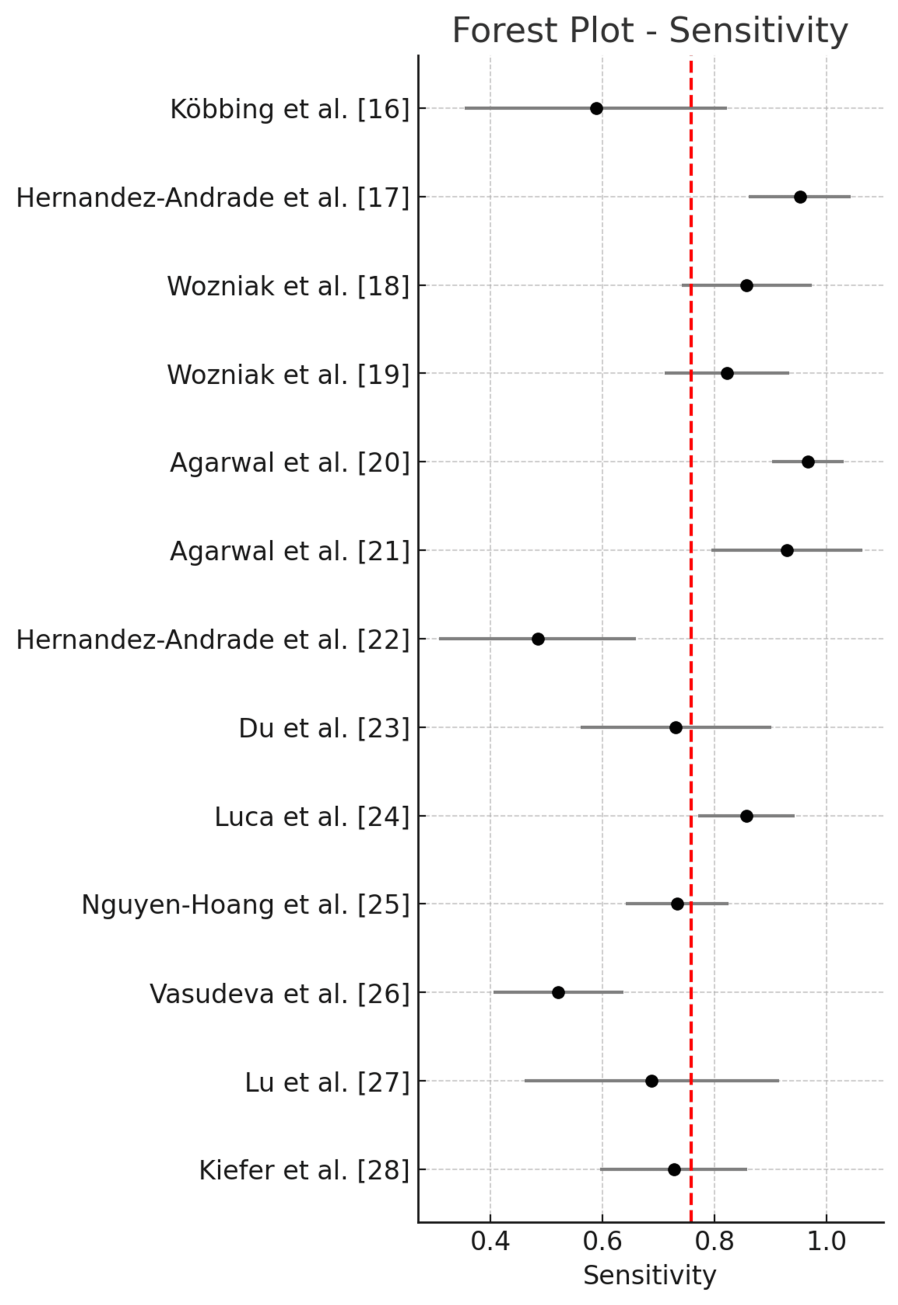
Forest plot of pooled sensitivity across all included studies.

Forest plot of pooled sensitivity across all included studies is illustrated in Figure 2. 

**Figure 3:**
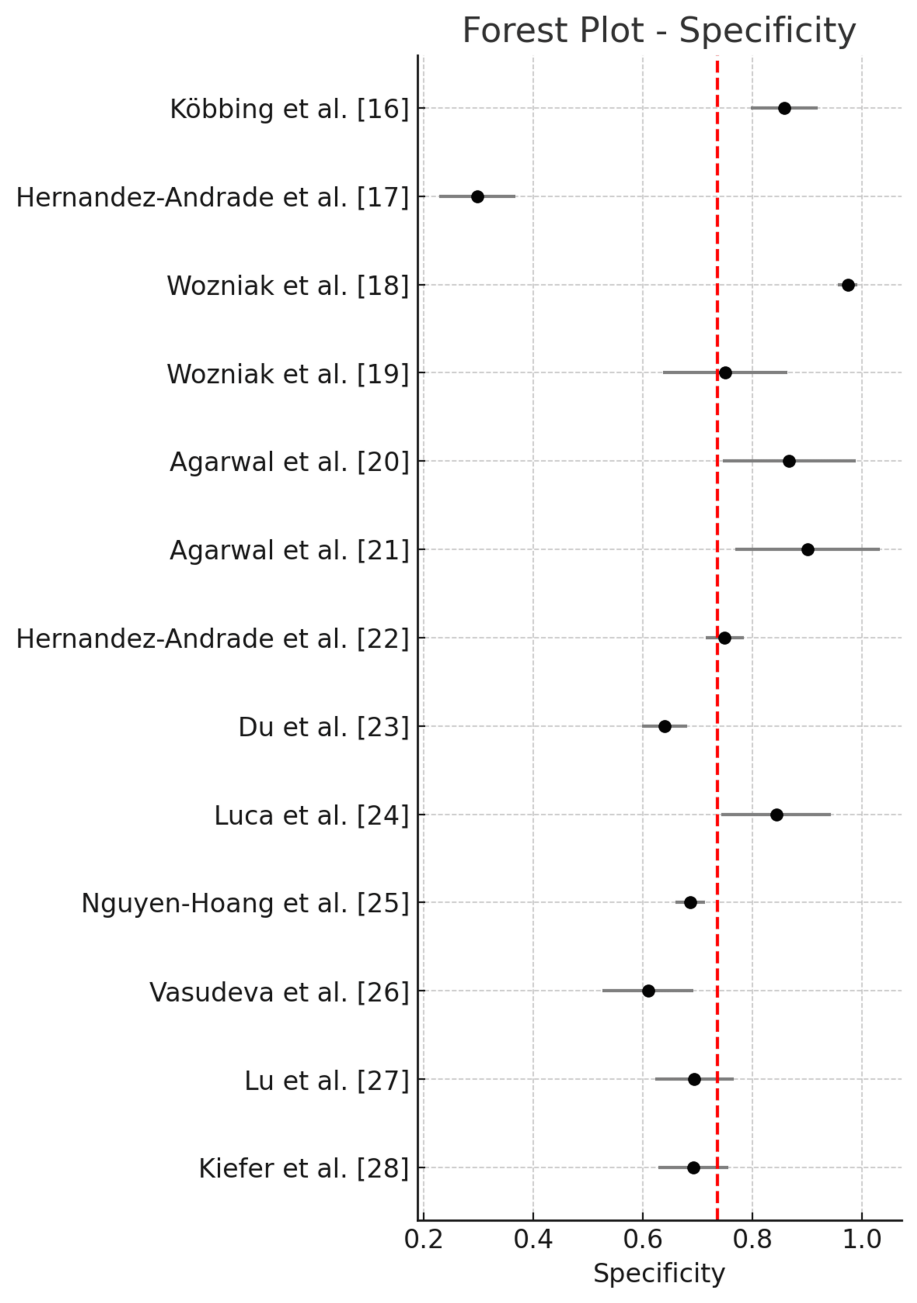
Forest plot of pooled specificity across all included studies.

Forest plot of pooled specificity across all included studies is illustrated in Figure 3. 

**Figure 4:**
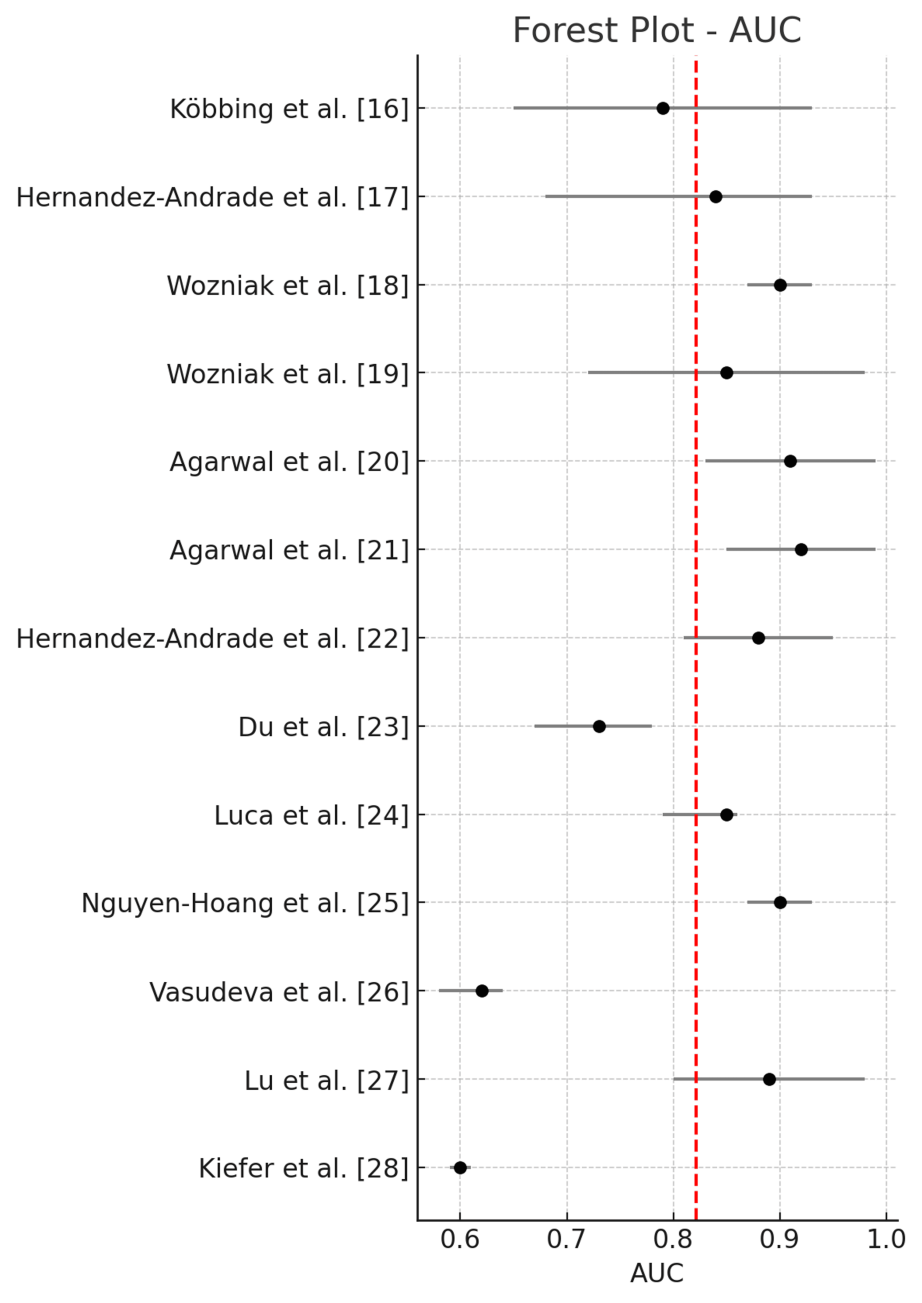
Forest plot of pooled AUC across all included studies.

Figure 4 illustrates the forest plot of pooled AUC across all included studies.

Subgroup Analyses

Subgroup analyses were deemed essential due to the substantial heterogeneity observed in the overall pooled results, suggesting important differences between the included studies. Factors such as elastography modality (strain vs. shear wave), population characteristics (symptomatic vs. asymptomatic), and gestational age at assessment (second vs. third trimester) are all likely to influence sensitivity, specificity, and overall diagnostic accuracy. By systematically examining these variables through subgroup analyses, we aimed to better understand the conditions under which the technique achieves its highest diagnostic performance. This approach also helps to identify specific populations and technical settings that yield the greatest predictive value, thereby supporting more informed clinical decision-making and enabling more tailored patient care.

Elastography Modality: Strain Elastography (SE) vs. Shear Wave Elastography (SWE)

In the subgroup analysis, SE studies demonstrated a pooled sensitivity of 0.773 (95% CI: 0.658-0.889, I² = 84.7%), pooled specificity of 0.710 (95% CI: 0.521-0.899, I² = 98.9%), and pooled AUC of 0.795 (95% CI: 0.689-0.901, I² = 96.9%).

SWE studies showed a pooled sensitivity of 0.766 (95% CI: 0.625-0.907, I² = 88.1%), pooled specificity of 0.740 (95% CI: 0.690-0.790, I² = 78.0%), and pooled AUC of 0.849 (95% CI: 0.682-1.016, I² = 99.1%).

Overall, SWE appeared to outperform SE in specificity, with a slightly higher AUC, suggesting superior diagnostic performance in predicting spontaneous preterm delivery. However, heterogeneity remained high within both subgroups, indicating that elastography modality alone does not account for the observed variability.

Symptomatic vs. Asymptomatic Populations

In symptomatic populations, pooled sensitivity was 0.913 (95% CI: 0.822-1.004; I² = 58.6%) and specificity was 0.835 (95% CI: 0.743-0.926; I² = 40.3%). In asymptomatic cohorts, sensitivity was 0.726 (95% CI: 0.628-0.824; I² = 83.1%) and specificity was 0.704 (95% CI: 0.586-0.823; I² = 98.8%). AUC estimates were 0.906 (95% CI: 0.858-0.955; I² = 0.0%) for symptomatic and 0.799 (95% CI: 0.695-0.903; I² = 98.9%) for asymptomatic groups. Heterogeneity was lower in symptomatic studies, suggesting that symptom status may influence both diagnostic performance and consistency of results.

Second Trimester Only vs. Third Trimester

Pooled sensitivity was 0.736 (95% CI: 0.648-0.825; I² = 81.5%) for the second trimester and 0.960 (95% CI: 0.902-1.018; I² = 0.0%) for the third trimester. Specificity was 0.708 (95% CI: 0.596-0.821; I² = 98.6%) and 0.882 (95% CI: 0.793-0.971; I² = 0.0%) for the second and third trimesters, respectively.

AUC was 0.803 (95% CI: 0.704-0.902; I² = 98.8%) in the second trimester and 0.916 (95% CI: 0.863-0.968; I² = 0.0%) in the third trimester. The lack of heterogeneity in third-trimester studies and significantly higher values across all metrics suggest that gestational age strongly influences diagnostic performance.

Across all subgroup analyses, diagnostic performance varied meaningfully by patient and methodological factors. Elastography mode analysis showed that SE achieved significantly higher specificity and AUC compared to SWE, while sensitivity was similar between modalities. Symptom status emerged as an important modifier: symptomatic women had markedly higher sensitivity, specificity, and AUC, with lower heterogeneity, indicating more consistent and accurate prediction of spontaneous preterm delivery in this group. Trimester of assessment also influenced performance, with third-trimester measurements showing substantially higher sensitivity, specificity, and AUC compared to second-trimester assessments, and virtually no heterogeneity, suggesting more stable diagnostic behavior later in pregnancy.

Taken together, these findings indicate that elastography’s predictive accuracy is not uniform across settings: optimal performance is observed when applied in symptomatic women in the third trimester using SE.

Subgroup analyses are displayed in Table 4.

**Table 4:** Meta-analytic summary estimates for diagnostic accuracy across predefined subgroups. CI: confidence interval, SE: strain elastography, SWE: shear wave elastography.

Covariates	Subgroup	Sensitivity (95% CI)	p (sensitivity)	I² Sens	Specificity (95% CI)	p (Specificity)	I² spec	AUC (95% CI)	p (AUC)	I² AUC
Elastography mode	SE	0.773 (0.658-0.889)	0.280	84.7%	0.710 (0.521-0.899)	<0.001	98.9%	0.795 (0.689-0.901)	<0.001	96.9%
Elastography mode	SWE	0.766 (0.625-0.907)		88.1%	0.740 (0.690-0.790)		78.0%	0.849 (0.682-1.016)		99.1%
Population	Symptomatic	0.913 (0.822-1.004)	0.000	58.6%	0.835 (0.743-0.926)	0.048	40.3%	0.906 (0.858-0.955)	0.000	0.0%
Population	Asymptomatic	0.726 (0.628-0.824)		83.1%	0.704 (0.586-0.823)		98.8%	0.799 (0.695-0.903)		98.9%
Pregnancy trimester	Second trimester only	0.736 (0.648-0.825)	<0.001	81.5%	0.708 (0.596-0.821)	<0.001	98.6%	0.803 (0.704-0.902)	<0.001	98.8%
Pregnancy trimester	Third trimester	0.960 (0.902-1.018)		0.0%	0.882 (0.793-0.971)		0.0%	0.916 (0.863-0.968)		0.0%

Leave-One-Out Analyses

To further explore heterogeneity and assess robustness, we performed LOO analyses for sensitivity, specificity, and AUC.

For sensitivity, the highest pooled estimate (0.798; 95% CI: 0.723-0.874; I² = 79.63%) was obtained when Vasudeva et al. [26] was excluded. The same study’s removal also yielded the lowest heterogeneity for sensitivity (I² = 79.63%).

For specificity, the highest pooled estimate (0.770; 95% CI: 0.677-0.862; I² = 97.86%) was observed when Hernandez-Andrade et al. [17] was excluded, whereas the lowest heterogeneity (I² = 94.53%; pooled specificity 0.710; 95% CI: 0.638-0.782) occurred upon removal of Wozniak et al. [18].

For AUC, the highest pooled value (0.839; 95% CI: 0.768-0.911; I² = 95.72%) was achieved when Kiefer et al. [28] was omitted.

In all cases, heterogeneity remained substantial, confirming that variability is not driven by a single study. Even after removing influential studies, I² reductions were minimal, suggesting heterogeneity is inherent to the dataset and likely reflects differences in study populations, designs, operator expertise, and elastography protocols.

Table 5 summarizes the LOO analyses.

**Table 5:** Summary of leave-one-out (LOO) analysis identifying study exclusions yielding the highest pooled estimates and lowest heterogeneity for sensitivity, specificity, and AUC. AUC: area under the curve.

Metric	Criterion	Study removed	Pooled	Lower 95% CI	Upper 95% CI	I² (%)
Sensitivity	Highest pooled	Vasudeva et al. [26]	0.798	0.723	0.874	79.63
Sensitivity	Lowest heterogeneity (I²)	Vasudeva et al. [26]	0.798	0.723	0.874	79.63
Specificity	Highest pooled	Hernandez-Andrade et al. [17]	0.77	0.677	0.862	97.86
Specificity	Lowest heterogeneity (I²)	Wozniak et al. [18]	0.71	0.638	0.782	94.53
AUC	Highest pooled	Kiefer et al. [28]	0.839	0.768	0.911	95.72

Discussion 

This systematic review and meta-analysis synthesized evidence from 13 studies published between 2014 and 2025 evaluating the diagnostic accuracy of cervical elastography for predicting spontaneous preterm birth. The pooled sensitivity of 77.1%, specificity of 73.3%, and AUC of 0.82 suggest that both SE and SWE provide moderate to good predictive performance, with a DOR of 11.05. Across the included studies, SWE tended to achieve higher sensitivity in later gestation, while SE often performed better in the early to mid-trimester. Importantly, some recent studies demonstrated that first-trimester SWE could detect cervical softening before any measurable cervical shortening, highlighting its potential role in identifying women at risk well before conventional ultrasound methods indicate a problem.

When compared with earlier reviews, our findings are broadly consistent with the meta-analysis by Wang et al. [12], who reported slightly higher pooled sensitivity and specificity of 84% and 82% respectively, with an AUC of 0.90 based on seven studies published up to 2018. That review also concluded that cervical elastography outperformed CL measurement in predicting preterm birth. The differences in pooled values between the two meta-analyses likely reflect our inclusion of a larger number of more recent studies that enrolled heterogeneous populations, including low-risk asymptomatic women, twin pregnancies, and women assessed as early as the first trimester. The narrative review by Gholamalipour et al. [29] emphasized the value of cervical elastography at 18-22 weeks in asymptomatic women, particularly for detecting abnormalities before clinical or sonographic evidence of cervical shortening. Our analysis supports this observation, while also extending the evidence base to show that diagnostic accuracy remains acceptable across a broader gestational range and with different elastographic techniques.

The implications of these findings are clinically significant. Cervical elastography appears to be a promising adjunct to CL measurement, offering the possibility of earlier and more accurate identification of women at risk for spontaneous preterm birth, even in populations where CL alone has limited predictive value. Early detection, especially during the first or early second trimester, could allow for targeted surveillance and timely preventive measures such as progesterone supplementation, cervical cerclage, or tailored antenatal care. The integration of elastography into clinical practice, however, will require standardization of acquisition protocols, region-of-interest definitions, and diagnostic cut-off values to reduce variability and ensure reproducibility.

This review has several notable strengths. It provides a comprehensive synthesis of literature spanning more than a decade, incorporating the most recent technological advancements in elastographic imaging. The inclusion of diverse populations and the exploration of subgroup analyses by technique, gestational age, and clinical risk status strengthen the generalizability of the findings. The statistical approach, including sensitivity analyses, adds robustness to the conclusions.

Nevertheless, limitations must be acknowledged. Statistical heterogeneity was high, with I² values exceeding 85% for sensitivity and over 98% for specificity and AUC, indicating substantial between-study variability. This heterogeneity likely arises from differences in study populations, ultrasound equipment, elastography algorithms, probe frequencies, region-of-interest placement, and cut-off thresholds, as well as the inherent operator dependency of strain elastography. Although subgroup analyses reduced heterogeneity in some comparisons, substantial variability persisted, underscoring the multifactorial nature of these differences and the urgent need for standardized measurement protocols.

Future research should focus on establishing internationally accepted technical standards for cervical elastography, including consistent cut-off values and acquisition procedures. Longitudinal studies are needed to track changes in cervical stiffness over the course of pregnancy and determine the optimal timing for measurement. The combination of elastography with CL, biochemical markers, and maternal history in integrated predictive models could further improve accuracy. As automated and AI-driven elastography analysis develops, there is an opportunity to reduce operator dependence and improve reproducibility. Cost-effectiveness analyses will also be important to determine whether this technology can be widely implemented, particularly in low-resource settings where preterm birth burden is highest.

## Conclusions

This systematic review and meta-analysis demonstrates that cervical elastography provides moderate to good diagnostic accuracy for predicting spontaneous preterm birth, with complementary strengths observed between strain and shear wave techniques across different gestational stages. While the technology shows promise as an adjunct to cervical length measurement, its clinical utility is currently limited by methodological heterogeneity, lack of standardized acquisition protocols, and variability in diagnostic thresholds. Standardization efforts, integration with established predictors, and the application of automated or AI-assisted analysis may enhance reproducibility and broaden clinical adoption. Ultimately, cervical elastography represents a valuable emerging tool for risk stratification in preterm birth prevention, but further large-scale, longitudinal, and methodologically rigorous studies are required before it can be routinely implemented in clinical practice.

## Appendices

Appendix 1 

**Table 6:** Quality assessment of the included studies.

Study	Patient selection (bias)	Patient selection (applicability)	Index test (bias)	Index test (applicability)	Reference standard (bias)	Reference standard (applicability)	Flow and timing (bias)
Köbbing et al. (2014) [16]	Low	Low	High	Low	Low	Low	Low
Hernandez-Andrade et al. (2014) [17]	Low	Low	High	Low	Low	Low	Low
Wozniak et al. (2014) [18]	High	Low	High	Low	Low	Low	Low
Wozniak et al. (2015) [19]	High	Low	High	Low	Low	Low	Low
Agarwal et al. (2018) [20]	High	Low	High	Low	Low	Low	Low
Agarwal et al. (2018) [21]	High	Low	High	Low	Low	Low	Low
Hernandez-Andrade et al. (2018) [22]	Low	Low	High	Low	Low	Low	Low
Du et al. (2019) [23]	Low	Low	High	Low	Low	Low	Low
Luca et al. (2023) [24]	Low	Low	Low	Low	Low	Low	Low
Nguyen‐Hoang et al. (2024) [25]	Low	Low	High	Low	Low	Low	Low
Vasudeva et al. (2024) [26]	Low	Low	High	Low	Low	Low	Low
Lu et al. (2024) [27]	Low	Low	High	Low	Low	Low	Low
Kiefer et al. (2025) [28]	Low	Low	High	Low	Low	Low	Low
